# Quantitative research on aesthetic value of the world heritage karst based on UGC data: A case study of Huangguoshu Scenic Area

**DOI:** 10.1371/journal.pone.0317304

**Published:** 2025-02-10

**Authors:** Xi Zhao, Kangning Xiong, Meng Zhang

**Affiliations:** 1 School of Karst Science, Guizhou Normal University/State Engineering Technology Institute for Karst Desertifcation Control, Guiyang, Guizhou, China; 2 Guizhou University of Finance and Economics, Guiyang, Guizhou, China; Shahid Beheshti University, ISLAMIC REPUBLIC OF IRAN

## Abstract

The World Natural Heritage is a rare and irreplaceable natural landscape recognized by all mankind, with outstanding significance and universal value. Among them, the World Heritage Karst sites(WHKs) holds an important position due to its special natural beauty and aesthetic value. In the field of landscape evaluation, interdisciplinary and interdisciplinary cooperation using different methods has always been a research focus. However, there is still a gap in the evaluation of natural landscape aesthetic value based on UGC(User Generated Content) data and deep learning models. This article is based on a public perspective, using social media UGC data, crawling images and texts as data sources, and combining SegFormer deep learning models, ArcGIS spatial analysis, natural Language Processing Technology (NLP) and other methods to conduct quantitative research on aesthetic value. Research has found that: (1) Huangguoshu Scenic Area has an excellent natural environment, and landscape elements with high naturalness (vegetation, water) are more attractive to tourists, with diverse landscape combinations; (2) There is no complete positive correlation between tourist sentiment bias, landscape diversity, and vegetation coverage. Emphasis is placed on the aesthetic perception path from bottom to top, from the surface to the inside. The comprehensive emotional value is 14.35, and the emotional values are all positively distributed. The distribution density and extreme value of positive emotions are greater than those of negative emotions; (3) The emotional bias of tourists is directly related to visual sensitivity, showing a synchronous trend of change. The visual sensitivity of the Great Waterfall and Dishuitan areas is relatively high, mostly at I-II level sensitivity. This method enhances the data source channel, which is conducive to obtaining the correct tourist evaluation orientation. In traditional subjective landscape evaluation, rational parameter indicators are added to reduce the probability of error, provide data support for its natural beauty description, break through the time and space limitations of aesthetic evaluation, and provide scientific reference for quantifying the aesthetic value of other heritage sites.

## Introduction

World Heritage (WH) encompasses cultural relics, historical sites, and natural landscapes that are recognized by UNESCO and the World Heritage Committee (WHC) for their rarity, irreplaceability, and universal value to humanity. These heritage sites offer unique economic, scientific, educational, recreational, and aesthetic benefits and hold significant importance for management purposes [1]. In the context of WH, "natural beauty" refers to the aesthetic value of natural heritage, describing the aesthetic quality of natural phenomena or regions [2]. According to Article 77 of the Guidelines for the Implementation of the World Heritage Convention, the WHC utilizes 10 precise and fixed criteria to assess and determine the importance of heritage sites and evaluate their Outstanding Universal Value (OUV). Notably, Criterion VII, which has undergone several revisions, is currently defined as sites that contain superlative natural phenomena or areas of exceptional natural beauty and aesthetic importance [3]. These frequent updates to Criterion VII indicate a growing stringency in the standards for natural beauty, making aesthetic evaluation a prevalent challenge in the heritage application process (Table 1). However, the application of Criterion VII ultimately makes it difficult to form a unique global standard, presenting regional differences [4]. This primarily stems from the fact that in WH aesthetic evaluations, influenced by the specific characteristics of the object assessed, the systematic appraisal of aesthetics constitutes a comprehensive cognitive process. Evaluators interpret and judge aesthetic objects based on their personal aesthetic experiences, emotions, and needs. This is a deeply intricate and complex psychological activity that cannot be entirely objective.

**Table 1:** Evolution of criterion VII.

Revision Date	Text
1977–1982	Criterion III contains unique, rare, or supreme natural phenomena, features or areas of exceptional natural beauty, such as ecosystems most important to humans, natural features (e.g., rivers, mountains, and waterfalls), spectacular scenes presented by large gatherings of animals, vast vistas covered by natural vegetation, and unique combinations of natural and cultural elements.
1983–1993	Criterion III contains the highest natural phenomena, forms, or features, such as outstanding examples of the most important ecosystems, areas of extraordinary natural beauty, or exceptional combinations of natural and cultural factors.
1994–2022	Criterion VII contains superlative natural phenomena or areas of exceptional natural beauty and aesthetic importance

Information from www.whc.unesco.org.

Therefore, based on the complexity of aesthetic evaluation and the improvement of public aesthetic awareness, scholars have expressed concerns about the objectivity and standardization of aesthetic evaluation, mainly including the following three aspects: (1) The openness of aesthetic evaluation system and the difficulty in coordinating the authority of evaluation standards: Analogies, which depend on expert knowledge and insights [5], primarily utilize qualitative analysis and lack quantitative metrics. This deficiency can lead to biased interpretations of Criterion VII across various cultural and social contexts. Consequently, this restricts the comprehensive and objective representation of aesthetic values in applications for World Heritage site(WHs) designation [6].Therefore, a single expert evaluation is not sufficient. As a special protected area, the WHKshas ecological sensitivity [7], and its protection and development require comprehensive consideration of multiple stakeholders [8]. The European Landscape Convention (ELC) emphasizes the central role of the public in landscape understanding [9]. However, cultural, social, and personal factors limit public participation, leading to differences in preferences for the same landscape [10,11]. This diversity can lead to conflicts between the openness of aesthetic evaluation systems and the authority of established standards. (2)The cognitive dimensions and understanding scope of aesthetic evaluation are limited: about 90% of human perception of the environment is obtained through visual senses, and visual perception plays a core role in landscape perception [12]. This dominance of visual perception has led aesthetic research to predominantly focus on visual aspects, emphasizing rational cognition and often addressing issues of natural beauty from an epistemological standpoint [13]. This approach sometimes conflates natural and artistic aesthetics. However, the methods for appreciating artistic and natural beauty differ substantially. Lörzing identifies four levels of human interaction with nature: the intervention layer, knowledge layer, perception layer, and interpretation layer [14]. Thus, the understanding of natural beauty should not be solely knowledge-based but should also incorporate physiological perception and psychological elements to create a multidimensional aesthetic perception system that comprehensively evaluates natural beauty.(3)Aesthetic value is difficult to quantify: Despite centuries of study in landscape aesthetics, standardized and quantitative evaluation methods remain elusive [15]. This is primarily because landscape evaluation emerges from the interaction between the objective environment and subjective perception [16], and bridging subjective perception with the objective environment poses a significant challenge [17]. The elicitation of beauty should not solely rely on objective stimulus parameters but should also consider subjective social constructs [18,19].

**Fig 1:**
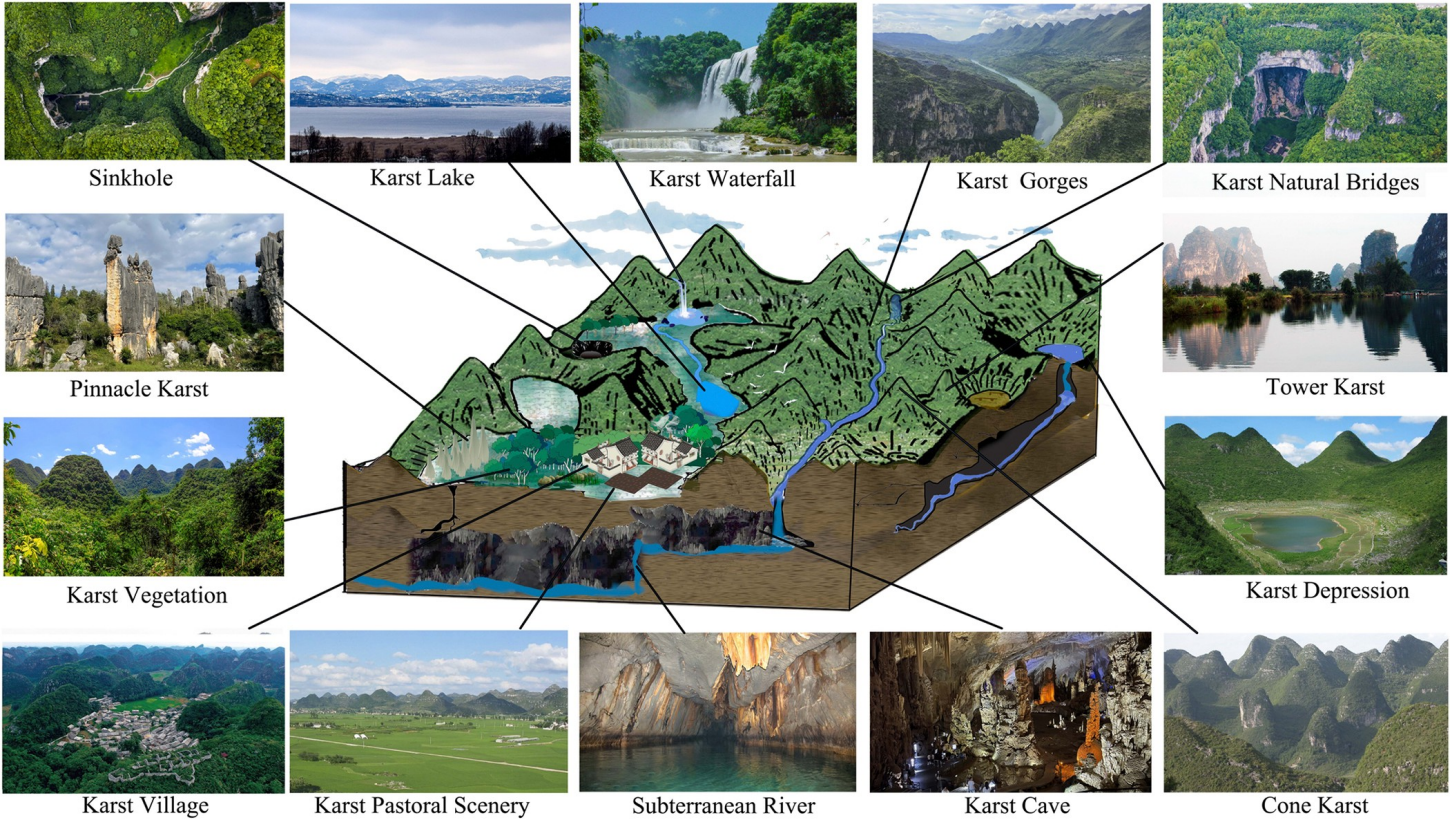
The karst region is endowed with a rich and unique landscape.

The natural beauty of the WHKs is attributed to its unique two-dimensional three-dimensional structure [20], which forms a diverse landscape of surface and underground [21,22] (**Fig 1**). Ultimately, it forms a landscape entity that resembles humans, gods, objects, flowers, birds, and animals, with various shapes and lifelike appearances, and has ornamental value and aesthetic enjoyment [23]. This has led to a gradual increase in the number of the WHKs. Since 1978, there have been a total of 38 WHs related to Karst, of which 25 Karst landscapes have been included in the World Heritage List(WHL) due to Criterion VII. In 2007, South China Karst series heritage sites used the analogy method to prove their aesthetic value when declaring, and had a wide impact worldwide, providing a standard demonstration for the declaration of other nominated sites [24]. Subsequently, analogy was widely used to demonstrate the uniqueness and distinctiveness of the aesthetic value of heritage sites [6,25–27]. However, evolving aesthetic perspectives have raised three key scientific issues, leading to growing concerns over the use of analogy. Recently, with the rapid advancement of information technology, fields such as neuroaesthetics [28], big data [29–31], and machine learning [32] have increasingly been applied in research. Digital technology facilitates data collection, recording, processing, and image recognition [33]. By leveraging big data, information technology [34], deep learning, and visual modeling [35], significant progress has been made in data collection, methodological innovation, and presentation of results. However, current applications of deep learning image segmentation techniques are primarily focused on medical and cultural landscapes, with a notable research gap in quantifying the aesthetic value of natural landscapes using big data-driven deep learning image segmentation techniques. Therefore, how to construct a comprehensive evaluation model of WHK aesthetic value and quantify its aesthetic value will be the focus of future heritage research [29], and an important component of achieving ecological protection and sustainable development.

The dissolution of soluble rocks by water in karst regions has given rise to a range of positive and negative topographies, resulting in a karst landscape with diverse morphologies. This distinctive geomorphology serves as the aesthetic carrier of the karst landscape.

This article embraces the concept of global localization, addressing three key scientific challenges in conjunction with the ’identification, evaluation, protection, and development’ framework for the natural beauty of the WHKs, and aligning with the global strategy to bolster nominations for WH status. Utilizing an interdisciplinary approach, this study harnesses UGC data, deep learning models, ArcGIS spatial analysis, and natural language processing technologies. These methodologies elucidate the components of aesthetic value in WHs from a data-driven standpoint, surmount the challenges associated with quantifying this value in karst landscapes, and specifically apply these insights to the Huangguoshu Scenic Area’s WH nomination. Consequently, this research provides a scientific foundation for the protection, development, and aesthetic evaluation of WHKs and other heritage locations.

## Materials and methods

### Study area

**Fig 2:**
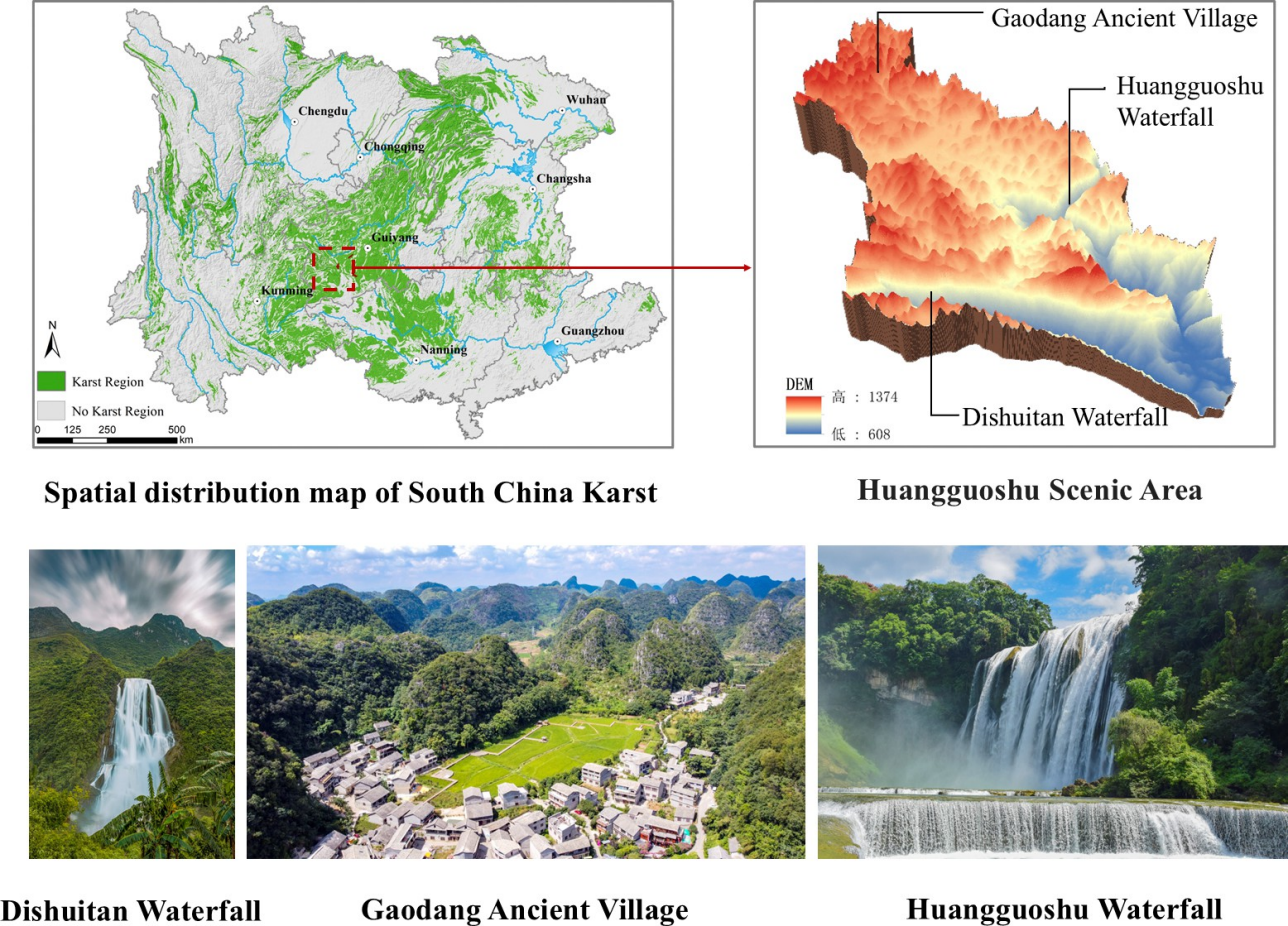
Location map of research areas.

The distribution area of karst landforms accounts for about 12% of the total global land area, primarily located in Southeastern Asia, the Mediterranean coast, Eastern Europe, the Middle East, the southeastern United States, and the Caribbean region. In China, carbonate rocks are extensively distributed, with a karst area of 3.443 million km^2^, making up about one-tenth of the national territory [36]. Among these, the ’South China Karst’ is the largest, most concentrated, and most typical landscape. Williams referred to the ’South China Karst’ and the ’Dinaric Karst’ as the world’s two most significant karst regions [21]. The Huangguoshu Scenic Area, situated in the western part of the Guizhou Plateau, mainly falls within the Huangguoshu National Scenic Area (Fig 2). It consists of three areas: the Great Waterfall Area (E105°40′05′, N25°57′08′), the Dishuitan Area (E105°36′21′, N25°59′29′), and the Gaodang Area (E105°40′25′, N26° 04′ 02′). This area consists of two distinct yet interconnected geographical units: the plateau and the canyon areas. These units vary considerably in their natural geography, geomorphic types, development, evolution, landscape characteristics, living environments, human habitation, and land use. The region boasts some of the world’s rarest natural landscapes and most pristine environments, holding an irreplaceable role as a prime representative of the South China Karst and global plateau mountains. In January 2019, the National Forestry and Grassland Administration of China approved the Huangguoshu Scenic Area’s application for inclusion in the World Natural and Cultural Heritage Project, designating a heritage site area of 34.70 km^2^ and a buffer zone of 112.30 km^2^.

### Methods

#### Text analysis method

The systematic extraction and analysis of text content using semiotics, structuralism, and linguistics analysis methods, in order to identify patterns, trends, or specific features, explore the structure and meaning of the text, and its research purpose is to use data to describe the inherent inclination and characteristics of the text’s expression content. This study combines text analysis with emerging technologies such as Natural Language Processing(NLP) and machine learning to deeply explore and analyze aesthetic expressions and evaluations from text data such as social media. Through quantitative methods, it reveals the aesthetic trends, preference differences, and factors affecting aesthetic judgments of different populations. Emotional analysis tools are used to determine the emotional tendencies of texts, such as positive, negative, or neutral, in order to clarify the emotional biases of different theme words and provide data support for understanding the aesthetic needs of the public.

#### GIS spatial analysis

GIS technology is a comprehensive tool specifically designed for efficient collection, storage, processing, analysis, and visualization of geospatial data. In this study, GIS technology was mainly applied to perform visual sensitivity analysis, which involves evaluating the degree of influence of various elements in the landscape on the observer’s visual perception. In addition, by using the Kriging interpolation method, research can spatially process UGC data to generate intuitive maps and charts. These visualization results help reveal the aesthetic characteristics and spatial distribution patterns of tourist experiences in karst landscapes. This method enhances the spatial analysis ability of the aesthetic indicator system and provides a scientific and intuitive means for evaluating the aesthetic value of the WHKs.

#### Image segmentation method based on SegFormer model

SegFormer is a semantic segmentation model based on Transformer, jointly proposed by researchers from NVIDIA and Peking University. Compared with other models, SegFormer has the following characteristics: (1) Efficiency: SegFormer is usually more efficient than other models because it uses a lightweight MLP decoder and the Transformer structure allows for better parallel processing; (2) Performance: SegFormer may provide better performance on certain datasets, especially in processing large-sized images and tasks that require capturing fine-grained features; (3) Generalization ability: SegFormer can benefit from pre trained Transformer models, which may provide better generalization ability and faster convergence speed; (4) Model size: SegFormer’s model size is usually smaller than other models, making it more suitable for deployment on resource constrained devices.

The selection of datasets is also crucial. Universal datasets are often not suitable for regions with highly differentiated karst landscapes. Therefore, self-labeled datasets can be designed to target specific features of karst landforms, ensuring that the dataset includes categories and characteristics essential for analyzing karst landscapes. Self-annotated datasets offer several advantages: (1) Higher annotation accuracy: Due to the complexity of karst landscapes, self-annotated datasets can ensure accurate recognition and annotation of each geological and topographic feature. This precise annotation is vital for enhancing the performance of segmentation models; (2) Dataset adaptability: They can be customized to meet specific research needs, such as selecting particular types of karst landscapes (e.g., caves, waterfalls, peak forests) or images captured under specific lighting and seasonal conditions; (3) Consideration of geographical and cultural backgrounds: Karst landscapes may vary across different regions, and self-annotated datasets can accommodate these geographical and cultural variations, thus better tailoring the model to specific regional applications; (4) Innovation in research: Employing self-annotated datasets can provide new perspectives and resources for research, which is significant for the advancement and dissemination of scientific knowledge. The annotated images include Huangguoshu, Jiuzhaigou Valley Scenic and Historic Interest Area, Libo, Xialongwan, Guilin Karst, and Shilin Karst, involving five categories and 19 subcategories under the theme ’mountain, water, forest, cave, people’ (Table 2). The model code can be found in Supporting information and is named: S1 File.

**Table 2:** Categories of image element tags.

ID	category	ID	category	ID	category
1	waterfall	7	cloth	13	ship
2	rivers	8	car	14	path
3	House	9	farmland	15	bridge
4	tree	10	mountain	16	blue sky
5	forest	11	stone	17	light
6	cave	12	people	18	rainbow
				19	mask

### Data sources and analysis

#### Data acquisition and indicator selection

This study primarily collected UGC data from social media platforms. UGC data refers to that users display their original content through the Internet platform or provide it to other users, which is diversified and personalized, real-time and interactive, rich and diverse. The emergence of UGC has enhanced the diversity and personalization of online content, offering innovative perspectives and approaches for researching traditional subjects using UGC. With the advancement of Web 2.0 and mobile internet technologies, UGC has emerged as a novel method for capturing tourists’ perceptions of destinations[37]. In the tourism sector, UGC is recognized as a valuable information source for National Tourism Organizations (NTOs), Destination Marketing Organizations (DMOs), policymakers, stakeholders, and prospective tourists. It includes genuine feedback from visitors to the destinations [38].

The selection of data crawling areas must be both representative and typical. The South China Karst region is recognized as one of the most emblematic karst landscapes globally. Professor Sweeting, renowned as the father of modern karst science, along with the geography department of Oxford University, visited Guizhou and Guangxi in 1986. He highlighted that many theoretical issues in global karst development could be addressed through research conducted in these areas. Similarly, Professor Ford Williams, a globally recognized karst scientist, after his 1989 research trip to Guizhou and Guangxi, affirmed that the South China Karst represents an exceptional landscape worldwide and should be prioritized as a WHs. Consequently, based on regional representativeness, data from tourist-shared texts and images from the Jiuzhaigou Valley Scenic and Historic Interest Area, Yunnan Stone Forest, Libo Karst, and Guilin Karst are considered. Additionally, for comprehensive data coverage, the marine karst at Vietnam’s Xialong Bay WHs is also included in the data collection scope. The platforms used for data crawling are Ctrip (https://you.ctrip.com), Two Steps Road (https://www.2bulu.com), and Six Feet (http://www.foooooot.com). Ctrip is a leading online travel service provider in China, serving over 90 million members with travel consultation services. Two Steps Road and Six Feet are the largest outdoor sports sharing platforms in China, enabling tourists from various countries and regions to share their outdoor travel experiences freely. The data collection period encompasses the maximum range searchable on these websites. Subsequently, duplicates, marketing advertisements, and garbled text are filtered out, yielding a total of 73,200 usable data entries.

All data used in this study are publicly available and collected according to the terms and conditions of the data provider. These data are anonymous and we have not collected any personal information. These data allow researchers to download and analyze them for scientific purposes, and therefore do not require ethical approval. The data analysis model used in the article is constructed based on existing and publicly available deep learning models.

Import the data into the text data obtained by the "Word Frequency Analysis" function of the ROST CM6 software, and standardize the vocabulary representing the same name and meaning. For example, use "color expression" to represent words such as "color", "deep blue", and "colorful", and use word segmentation and stop word lists to filter out high-frequency vocabulary such as "5 hours" and "6 kilometers" that do not refer to the analysis target and are unclear, Finally obtaining the top 30 high-frequency vocabulary in the ranking (Table 3).

**Table 3:** Ranking and classification of high-frequency words based on UGC data.

ID	Words	Frequency	category	ID	Words	frequency	category
1	wonder	2824	visual/unique	16	history	703	psychology
2	changing	1948	diversity	17	vegetation	684	naturalness
3	colour	1425	vision	18	vitality	665	psychology
4	it seems	1254	psychology	19	shape	608	diversity
5	locality	1121	psychology	20	nature	601	naturalness
6	unique	1083	unique	21	richness	589	diversity
7	grotesque peak	1076	vision	22	peaceful	570	psychology
8	spectacular	1007	psychology	23	original	475	naturalness
9	water	893	vision	24	roar	456	hearing
10	weather	855	touch	25	deep and serene	437	naturalness
11	continuous	835	vision	26	close	342	vision
12	beautiful	817	psychology	27	free	406	psychology
13	rocks of grotesque shapes	756	diversity	28	be worth	361	psychology
14	cave	741	diversity	29	broad	342	vision
15	seasons	722	diversity	30	wonderand	247	psychology

Aesthetics encompasses emotional, material, and spiritual dimensions of beauty, which are interconnected. The most sublime expression of beauty emerges when these three dimensions are unified [39]. Based on the above table, it is clear that the public perspective aesthetic indicators are concentrated in five aspects: naturalness, diversity, physiological perception, psychological perception, uniqueness, etc. Combined with the existing conditions of deep learning models and previous research, the final indicators are determined as follows:

Diversity: Landscape diversity is often used to evaluate the aesthetic perception of different stakeholders [40,41]. Studies have shown that landscape diversity is a key determinant of landscape beauty, reflecting the structural characteristics of ecosystems [15]. The diversity of karst landscapes is a core part of their aesthetic value. Due to their unique geological processes, karst terrain has formed rich and varied geomorphic features, such as karst caves, stalagmites, sinkholes, underground rivers, and peak clusters. The diversity of these landforms not only provides visually spectacular and diverse natural landscapes, but also reflects the evolution of Earth’s history and the complexity of geological processes.

Vegetation coverage: Naturalness has always been an important factor in landscape evaluation [42]. Naturalness reflects the original state and undisturbed natural processes of karst landscapes, and the most intuitive manifestation of naturalness from the perspective of tourists is vegetation coverage. Vegetation coverage is used as the data support for landscape evaluation [43], and naturalness represented by vegetation coverage is a key factor in evaluating the natural beauty of karst areas. It not only reflects the integrity and stability of the ecosystem, but also directly affects the visual appeal and biodiversity of the landscape.

Visual sensitivity: Approximately 90% of human perception of the environment is obtained through visual senses, and visual perception plays a core role in landscape perception [12]. Visual sensitivity has been used in landscape evaluation [44,45].High visual sensitivity areas often become the focus due to their unique terrain, vegetation configuration, or lighting effects.

Psychological perception: In the relationship between natural beauty and natural aesthetics, with the transformation of the concept from "beauty" to "aesthetics", the focus of natural beauty is also shifting from the objective essence of beauty to the psychology of the aesthetic subject at a deeper level [46]. Psychological perception evaluation significantly reflects the personal experience and emotional response of tourists to karst landscapes, revealing their preferences, interests, and satisfaction. Insight into the psychological perception of tourists is essential for identifying and maintaining landscape elements that have profound psychological and emotional significance to the public, helping to protect and develop heritage sites.

### Quantitative evaluation of aesthetic value based on UGC data

1) Determination of research site

**Fig 3:**
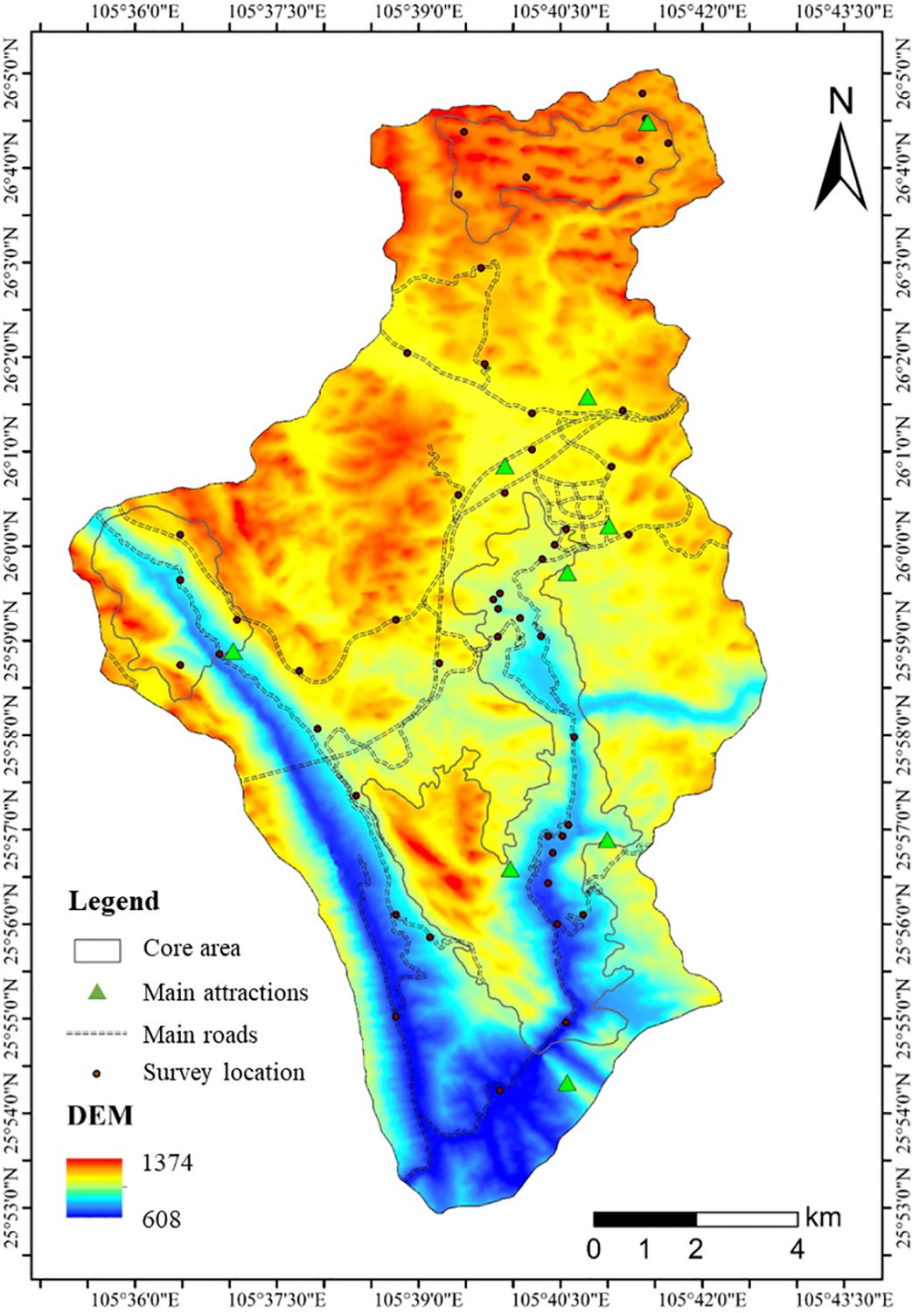
Distribution of survey points.

Extract the POI of the obtained photos, import the longitude and latitude into ArcGIS 10.2, and determine 50 survey points based on the density of tourist interest points and expert opinions. Among them, there are 28 survey points in the core area and 22 survey points in the buffer zone (Fig 3).

2) Quantifying the Aesthetic Value of Images

**Fig 4:**
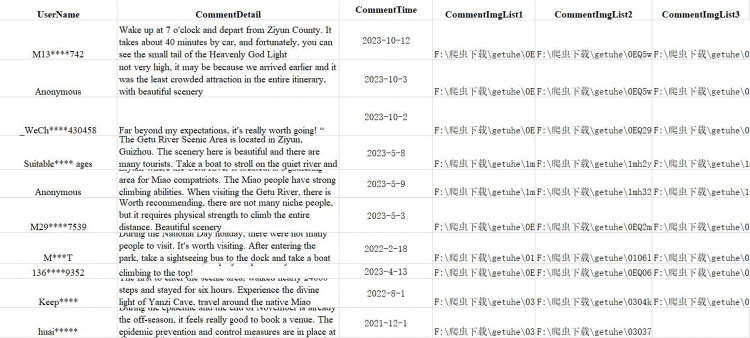
Part of the crawl content display.

The UGC data in this article mainly consists of evaluation texts and images in the study area; Using automation and API excuses to crawl Ctrip(https://you.ctrip.com),Two step road(https://www.2bulu.com/) and Six feet(http://www.foooooot.com/).The website’s POI photos, text, user ID, and upload time information (Fig 4), and the final crawling situation are shown in Table 4.

**Table 4:** UGC climbing statistics in HuangGuoshu scenic area.

Website	Ctrip		Two steps		Six feet	
**Category**	Images	Text	Images	Text	Images	Text
**Number**	8952	55232	1023	20236	754	15786

3) Quantifying the Value of Text Aesthetics

Although photos of scenic spots uploaded by tourists can capture their focus and suggest specific preferences, emotional tendencies are complex psychological states influenced by multiple factors such as personal emotions, experiences, and expectations. These states make it difficult to fully reveal them solely through photos. The content of photos may also be affected by technical factors such as shooting techniques and post-processing, which in turn can influence the evaluation of emotional tendencies. To accurately grasp the emotional tendencies of tourists, it is necessary to incorporate text analysis, and this study primarily utilizes sentiment analysis methods. Using the WeChat Cloud SnowNLP sentiment analysis software, we employ a vast and authoritative sentiment dictionary, along with the inclusion of degree words and negation words, and combine multiple dimensions such as context to determine emotional tendencies. By setting emotional words, customizing dictionaries, defining synonyms, and other steps for sentiment analysis, the standard for setting emotional words requires setting emotional words and scores. The format is: word = value (range -3 to 3, negative when value is less than 0, positive when value is greater than 0), word length must be > = 1, multiple words separated by commas. Below are examples of emotional words (Table 5).

**Table 5:** Setting of emotion words.

Sentiment Word	Magnificent	shocking	elegant	peaceful	crowded	dirty	commercialized	destroyed
Score	3	3	2	1	-1	-2	-2	-3

#### Visual sensitivity analysis

Approximately 90% of human environmental perception is obtained through visual senses, and visual perception plays a core role in landscape perception 12]. IUCN recognizes that the visual factors of landscape are an important component of natural value [47]. Consequently, the study of a landscape’s aesthetic value is inherently linked to the assessment of visual perception. Visual Sensitivity Analysis (VSA) is employed to determine the sensitivity of various observers to landscape resources at specific spatial scales, providing essential data for landscape planning, protection, and management. The use of ArcGIS for visual sensitivity research has become the main method. Based on previous research, this article divides field of view analysis into three aspects: relative slope, relative field of view, and probability of occurrence [48–50].The relative slope can affect our spatial perception of the landscape, such as the steepness or flatness of the mountain; Relative distance can affect our perception of the size and depth of the landscape, such as the size and proximity of distant mountains; The probability of occurrence can reflect the rarity and uniqueness of the landscape, such as the frequency of appearance of a specific landscape element (such as waterfalls, ancient trees, etc.). These visual sensitivity factors can all affect our evaluation and perception of landscape aesthetics. Visual sensitivity is composed of relative slope (Table 6), relative distance (Table **7**), and probability of occurrence (Table **8**), which ultimately add up to the overall visual sensitivity (Table **9**).

**Table 6:** Classification of relative slope sensitivity.

α	0°<α<15°	15°<α<30°	30°<α<45°	>45°
**Sensitivity**	Low	Ordinary	Medium	High

**Table 7:** Details of relative distances and observations.

Relative distance	Observation details
**0-2m**	Fine textures such as dissolution marks, calcification, cave sediments, etc
**2-50m**	Structural texture, internal texture
**50-100m**	Overall contour and surface texture
**100-500m**	External contours and spatial relationships with surrounding landscape
**>500m**	The physiological perception of general landscapes becomes blurred

**Table 8:** Landscape view degree table of Huangguoshu scenic.

Accumulated visual field degree	value	count	Area of view
**Lowest**	0–48	861466	91.6325%
**Low**	48–96	74605	7.9356%
**Average**	96–144	3791	0.4032%
**Slightly high**	144–192	266	0.0283%
**Highest**	192–240	4	0.0004%

**Table 9:** Classification of visual sensitivity levels.

Comprehensive sensitivity	Score	Relative inclination	Score	Relative distance	Score	Probability of occurrence	Score
**Level I**	10–12	0–15°	4	0-2m	4	High	4
**Level II**	7–9	15–30°	3	2-50m	3	Average	3
**Level III**	4–6	30–45°	2	50-100m	2	Low	2
**Level IV**	1–3	>45°	1	100-500m	1	Lowest	1

## Result

### General characteristics

**Fig 5:**
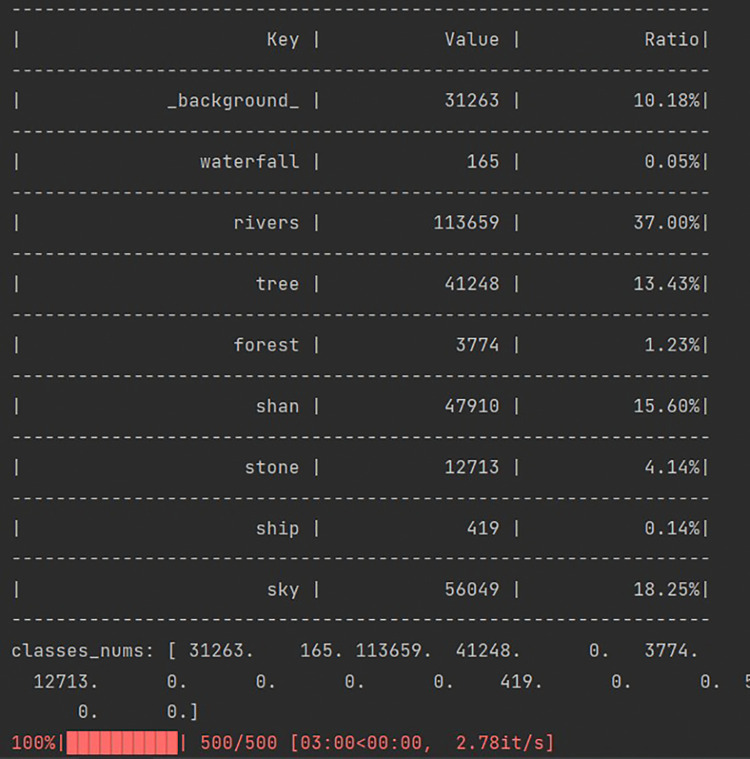
Statistics result. The statistical results include three items: Key, Value, and Ratio. Key represents the landscape element identified in each image, Value represents the quantity of the element, and Ratio represents the proportion of the element.

The content analysis of photos can provide a very detailed depiction of the scenery that tourists appreciate [51], and the interpretation of photos depends on the content captured in the photos[52]. By analyzing the photos of the photographer, their aesthetic perception and experience can be explored [53]. Using the SegFormer model, randomly select 500 images from the obtained photos multiple times, perform semantic segmentation on the images, and clarify the classification and proportion of each landscape element. The output result is shown in the following Fig 5.

After multiple tests, the percentage of the main components of the landscape elements in the three areas was finally obtained based on the number of pixels (top 8), and the average of multiple experiments was taken as the result, as shown in Table 10:

**Table 10:** Distribution of main elements in Huangguoshu.

Category	Vegetation	Mountain	Water		Stone	Sky	Cave	Path	People
**Number**	33.68%	28.36%	6.32%	2.34%	21.02%	9.55%	4.52%	0.42%	0.44%

Note: After on-site investigation and photo inspection, it was found that the mountains in this area have vegetation, so the true value of vegetation is equal to the mountain+vegetation close-up.

**Fig 6:**
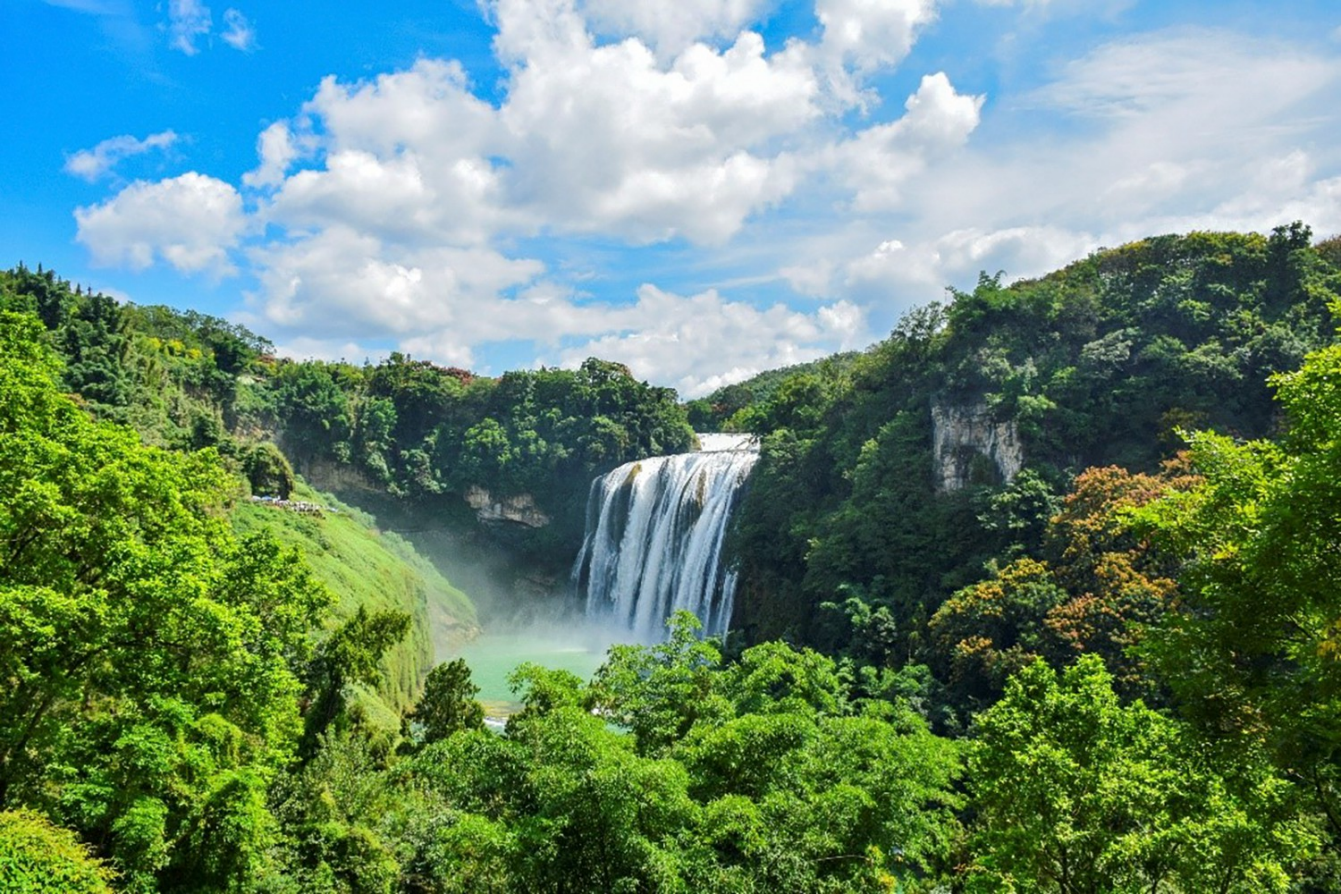
Combination of Huangguoshu landscape elements.

In the image segmentation of Huangguoshu, 16 natural elements were identified, indicating that in the Huangguoshu scenic area, the main categories of natural elements are relatively concentrated, accounting for a significant portion of tourists’ visual experience, reaching 77.43%. By performing fine image segmentation on karst landscape photos, we acquired data that delineated the compositional characteristics of the landscape. Vegetation covers about one-third of the image area (33.68%), indicating that natural plants in the landscape grow abundantly, providing a rich ecological environment and green background for karst areas. The mountain occupies 28.36% of the proportion, highlighting the main characteristics of karst terrain, which is a unique landform formed by long-term erosion and dissolution of soluble rocks such as limestone. Although rivers (6.32%) and waterfalls (2.34%) account for a relatively small proportion in the overall landscape, they are key factors in the formation of karst landforms, adding dynamic hydrological features and visual appeal to the landscape. Rocks account for 21.02%, reflecting the characteristics of exposed rocks in karst areas. These rocks have undergone thousands of years of weathering, forming unique landforms and are valuable resources for exploration and research. The blue sky accounts for 9.55%, providing a broad and fresh view of the karst landscape, forming a sharp contrast with the terrain on the ground, and enhancing the sense of hierarchy of the landscape. Roads only account for 0.42%, which may indicate that the natural environment in the region remains relatively primitive, and human activities have a relatively small impact on the natural landscape. Caves comprise 4.52% of the total landscape and, as a typical feature of karst landforms, they serve not only as natural laboratories for geologists but also as mysterious and intriguing destinations for tourists. Human activity, accounting for only 0.44% of the landscape, is almost negligible, suggesting that the scenic area is either largely undeveloped and well-preserved or that the photographs were taken from angles that avoid depicting crowds. Overall, this data set depicts a karst landscape predominantly shaped by natural elements with minimal human impact. Each category showcases its own natural beauty and geological significance, together creating a diverse and harmonious ’mountain-water-stone-forest’ environment (Fig 6).

**Fig 7:**
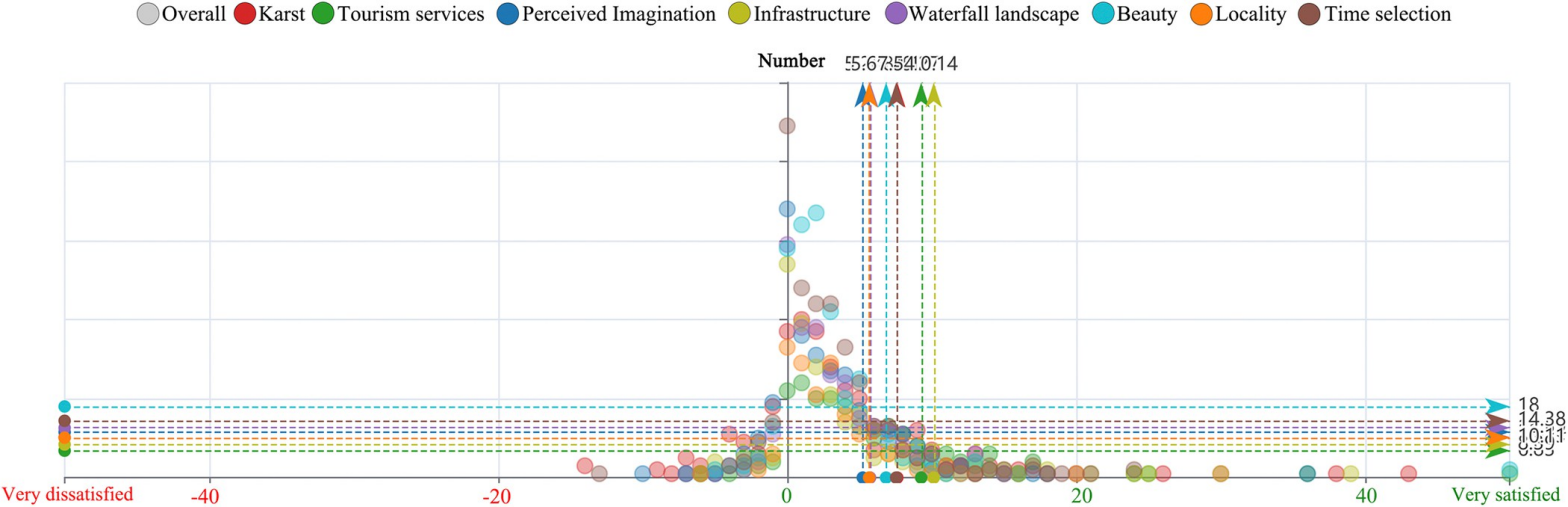
Overall emotional value and quantity distribution of Huangguoshu.

Subsequently, the photo text was explored, and the results are shown in the Fig 7.

Specifically, negative emotions are mainly concentrated within the range of 0 to -20 points, especially with a more uniform distribution around -10 points. Positive emotions tend to cluster within a range of 20 to 40 points, with extreme values even exceeding 40 points, with an average emotional value of 14.65. This further confirms the overall positive sentiment of tourists towards the Huangguoshu landscape. The emotional values of different theme words vary, with infrastructure ranking highest at an emotional value of 10.14. This indicates that the infrastructure of the Huangguoshu Scenic Area is well-developed, satisfying the aesthetic needs of tourists and earning high praise. The emotional values of theme words such as tourism services, karst formations, time selection, beautiful scenery, waterfall landscapes, locality, and perceived imagination are sequentially arranged. All demonstrate that tourists have high satisfaction with these key thematic elements, with an average emotional value exceeding 5 points. Generally, positive emotions predominate over negative ones.

### Quantification of aesthetic value of sub indicators

**Fig 8:**
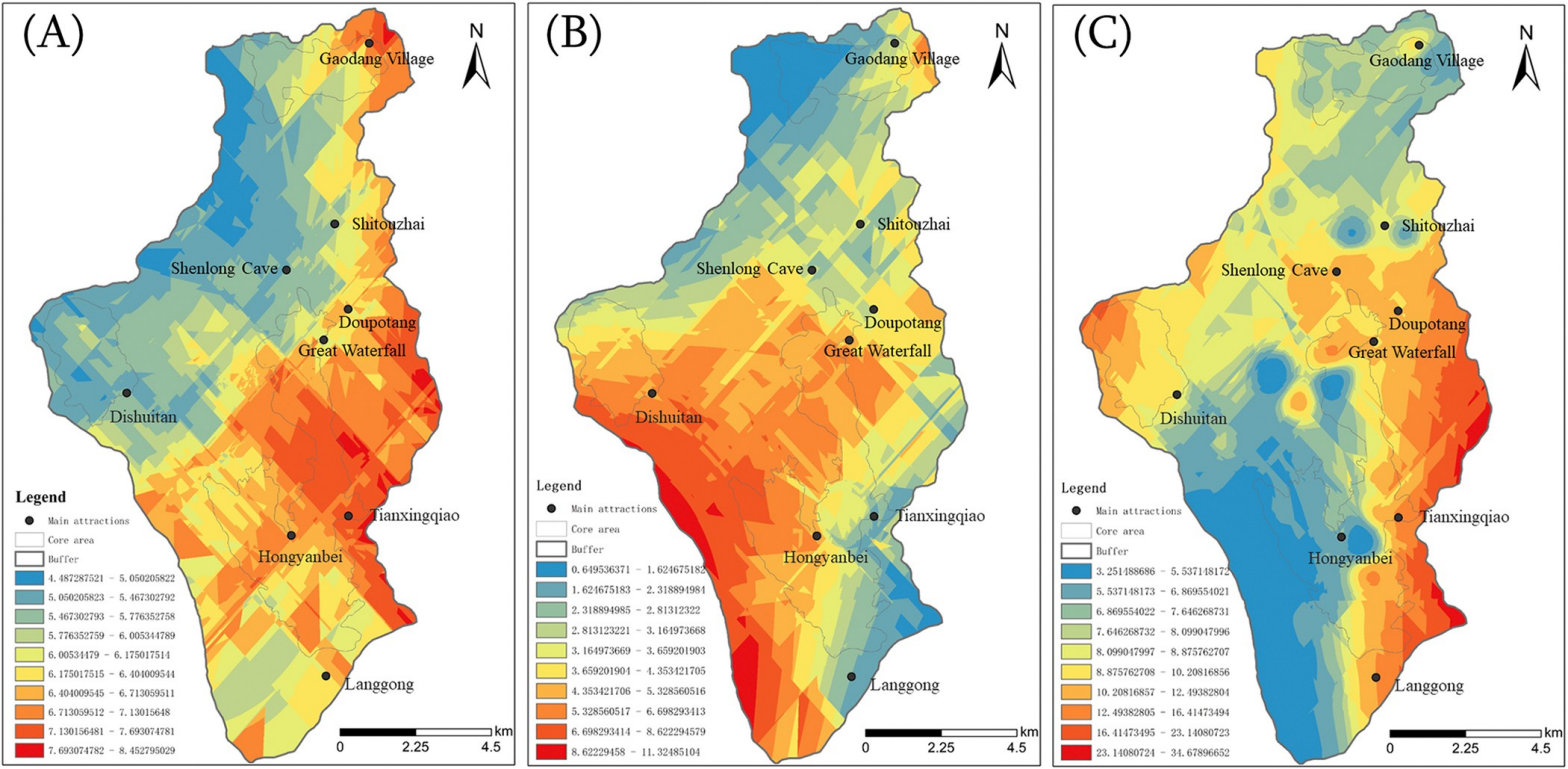
Visualization of sub-index results. A: landscape diversity distribution, B: vegetation coverage distribution, and C: emotion value distribution.

Subsequently, the aesthetic value represented by the sub-indicators was quantified and analyzed. Combining the above methods and indicators, the landscape diversity, vegetation coverage, and emotional values of each point were calculated based on UGC data. Some locations had limited access for tourists due to undeveloped areas and environmental protection concerns, resulting in scarce UGC data. Consequently, the team conducted field investigations and incorporated expert assessments to construct indicator values for these locations. The Kriging interpolation method was used for visualization, and the results are displayed in Fig 8.

Analysis shows that among the three areas of Huangguoshu Scenic Area, the landscape diversity is highest in the Great Waterfall area, followed by the Gaodang area, and lowest in the Dishuitan area, showing an overall decreasing distribution pattern from right to left. Especially in the Great Waterfall area, the Tianxing Bridge landscape integrates various landscape elements such as "mountains, water, rocks, forests, and caves", exhibiting the richest distribution of elements. According to the analysis of UGC photos, the overall vegetation coverage shows a distribution trend of more at the bottom than at the top, with the Dishuitan area slightly higher than the Great Waterfall area, and the Gaodang area having the lowest vegetation coverage. The distribution order of emotional values is the highest in the Great Waterfall area, followed by the Dishuitan area, and the lowest in the Gaodang area. This result indicates that there is not a complete positive correlation between tourist emotional responses and landscape diversity and vegetation coverage.

**Fig 9:**
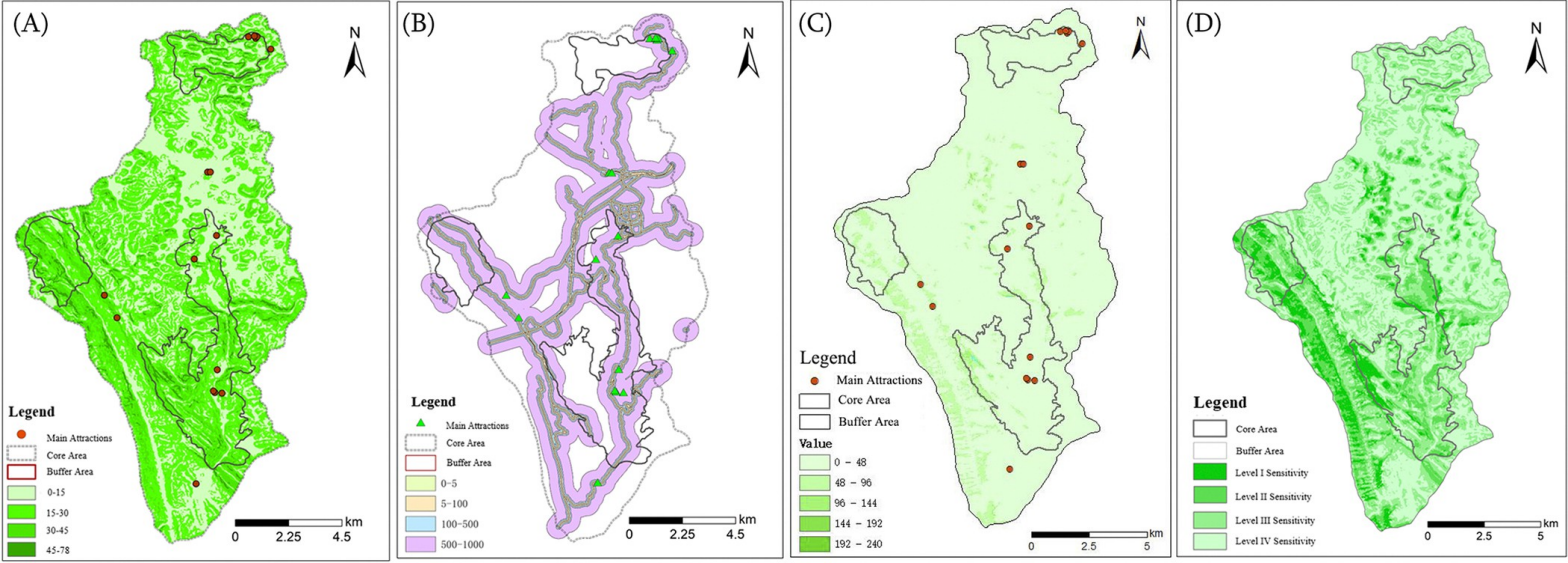
Visual sensitivity results visualization. A: relative slope, B: relative distance, C: visual probability, D: comprehensive sensitivity.

The visual sensitivity results show (Fig 9) that in the Huangguoshu area, the Huangguoshu Waterfall, Steep Slope Pond Waterfall, Silver Chain Falling Pond Waterfall, Dishuitan Waterfall, and Tianxingqiao Scenic Area have a medium to high visual sensitivity to the slope, while the Shitouzhai and Gaodang Scenic Area have a moderate slope sensitivity. The reason is that the village gathering areas are mostly in flat and open areas, and the visual impact brought by the slope tends to be average. In the Huangguoshu area, the main scenic spots are all within observable range. The relative distance between the Great Waterfall and the Dishuitan Waterfall is within 100-500m, allowing for clear observation of the outer contour of the waterfall and its spatial relationship with surrounding landscape elements. The steep slope pond waterfall and the silver chain falling pond waterfall are within 50-100m, while the Shenlong Cave, Shitouzhai, and Gaodang ancient architectural complex in the Tianxingqiao scenic area are within 0-5m, allowing for close observation of details. Finally, the slope, visibility, and video rate mentioned above are superimposed according to Table 7 to obtain a comprehensive sensitivity. Visual sensitivities such as the Great Waterfall and Dishuitan are relatively high, mostly at I-II levels, and should be strictly protected to reduce development and avoid the destruction of their aesthetic value. However, visual sensitivities in high rise areas are relatively low, and infrastructure should be optimized to scientifically enhance aesthetic value.

## Discussion

**Fig 10:**
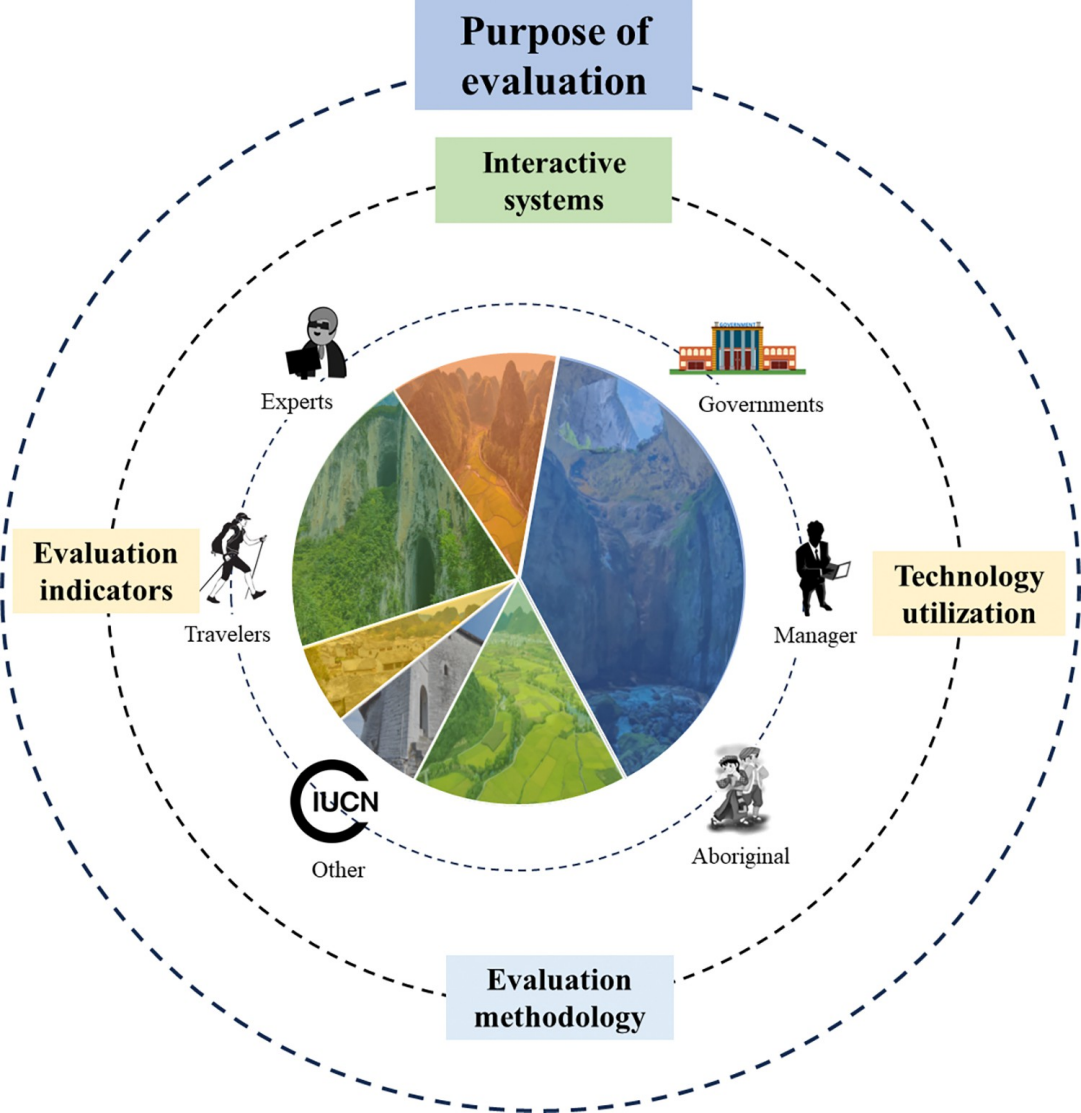
Participation of Different Stakeholders in the Aesthetic Evaluation System of the WHKs.

**Fig 11:**
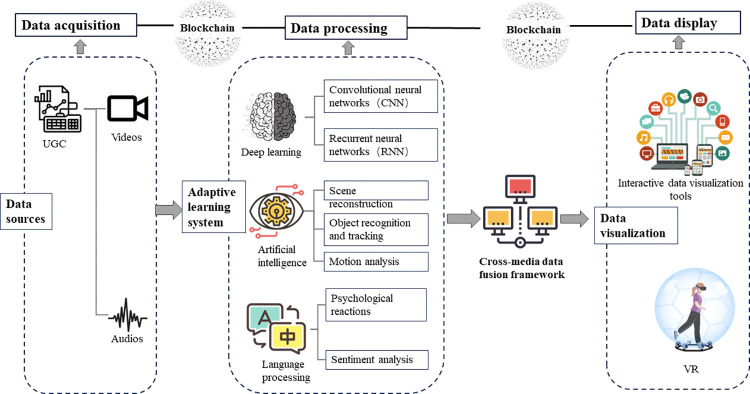
Using multivariate UGC data for aesthetic evaluation.

This study only quantifies the aesthetic value of some the WHKs aesthetic indicators from the perspective of UGC data, explores the aesthetic experience from the perspective of tourists, and has not yet formed a unified aesthetic indicator system. Building a comprehensive dynamic evaluation system still requires interdisciplinary and multi domain collaboration in the future, and the following aspects of research should be strengthened in the future:

1) Strengthen the construction of basic aesthetic databases: Building a comprehensive and detailed basic aesthetic database is crucial for evaluating the aesthetic value of natural landscapes, especially in areas with unique geographical features such as karst. At present, there is a significant data gap in the natural element database that deep learning image segmentation technology relies on, which limits the precise capture and evaluation of specific natural landscape aesthetic characteristics. For this purpose, the system needs to collect image data containing multi-dimensional information such as terrain, vegetation, and hydrology, and record from multiple angles under different seasons and conditions. The collaboration of interdisciplinary experts is crucial for ensuring the scientific and systematic nature of data, which will provide a solid data foundation for the protection, planning, and precise training of artificial intelligence algorithms for natural landscapes.

2) Improve the selection of multidimensional indicators: (1)Political, cultural, regional, geographical and other factors have an impact on heritage landscapes, and make the landscape and environment adapt to each other [54]. This impact is presented through different stakeholders. In the context of multicultural integration, it is crucial to cover the aesthetic preferences of various stakeholders. Experts and scholars tend to focus on the scientific value of landscapes, such as formation mechanisms, ecological structures and functions, biodiversity, and are committed to promoting landscape conservation through research. Managers focus on the tourism potential of landscapes, exploring strategies for resource development, providing high-quality services, and promoting tourism protection. Indigenous communities place greater emphasis on the social benefits of landscapes, caring about their impact on the local economy, quality of life, and ways to participate in conservation. Tourists focus on the natural beauty of the landscape and related psychological experiences. Building a comprehensive communication platform from various perspectives, utilizing technological means, with the aim of considering the interests and needs of all parties mentioned above, to ensure fairness and balance in evaluation (Fig 10); (2)Considering the selection of indicators from three aspects: WH standards, ecological environment conditions, and aesthetic experience; the WH standard indicators, including factors such as the uniqueness, authenticity, and integrity of heritage sites, ensure that the evaluation system is in line with international protection standards. Ecological environment status indicators, involving biodiversity, ecosystem health, and sustainability of natural resources, reflect the environmental quality and ecological value of heritage sites. Aesthetic experience indicators, which cover tourists’ sensory experiences, cultural perceptions, emotional responses, etc., depict the aesthetic appeal and cultural significance of heritage sites. The selection of these three indicators aims to form a multidimensional and interdisciplinary evaluation framework to comprehensively and deeply evaluate the aesthetic value of the WHKs.

3) Incorporating multivariate data to construct a dynamic evaluation system: Unlike traditional questionnaire surveys of tourists, which can be limited and influenced by participants’ willingness to respond, the vast and open nature of cyberspace offers a more accurate reflection of tourists’ genuine perceptions of landscape aesthetics. This approach significantly reduces research costs and enhances the scientific validity and practicality of the studies [55]. UGC data types extend beyond images and text. The popularity of short video platforms has mainstreamed video and audio content, turning them into key data sources (Fig 11). These multimedia data provide richer informational dimensions. Videos capture both the dynamic changes in landscapes and time-related aesthetic elements, while audio tracks contribute data on environmental and natural sounds. Analyzing video dynamics can uncover the changing aesthetic features of natural heritage sites. Similarly, studying audio textures and rhythms can reveal sound-based aesthetic characteristics that static images and text cannot. Consequently, future research should focus on effectively leveraging various UGC data forms, developing comprehensive data processing and analysis methods, constructing an extensive and dynamic multidimensional aesthetic recognition and evaluation system, and applying multi-source data to quantitatively study aesthetic values.

4) Clarifying the aesthetic carrier and promoting the coordinated development of protection and tourism: The goal of identifying the aesthetic carrier and assessing its aesthetic value is to foster the harmonious protection and development of the WHKs. The aesthetic value of these heritage sites should be categorized into direct value, potential value, and background value. The direct value is evident in tourism revenue and branding impact, and should be a primary focus in tourist areas. The potential value, linked to ecological conservation and educational research, should be moderately developed in buffer zones. The background value, associated with local culture, historical heritage, and spiritual life, ought to be primarily preserved in the core areas. To develop alternative tourism, emerging technologies such as Story Map should be employed [56], and other advanced technologies like Augmented Reality (AR), Virtual Reality(VR), and Unmanned Aerial Vehicles(UAV) should be utilized to more effectively protect and enhance the WHKs.

## Conclusions

This study utilizes UGC data, employing text and images for indicator selection, evaluation processes, and interpretation of results. By integrating UGC data with these indicators, we can thoroughly explore the underlying value of the data, overcoming the temporal and spatial limitations inherent in traditional evaluations. This approach allows researchers to derive richer and more profound insights from a substantial volume of authentic data, thus facilitating more objective and varied evaluations of aesthetic values. Research has found that: (1) The Huangguoshu Scenic Area has an excellent natural environment, with 16 natural elements discovered. Vegetation (33.68%) and mountains (28.36%) are the most prominent features of the landscape, displaying rich ecology and undulating terrain. The significant proportion of rocks (21.02%) highlights the typical karst landforms in karst areas. Rivers (6.32%) and waterfalls (2.34%), although relatively small, add vivid hydrological features to the landscape. Caves (4.52%) lend a sense of mystery to the terrain. The blue sky (9.55%) provides a broad background for the landscape, while the minimal presence of roads (0.42%) and people (0.44%) indicates a limited impact from human activities, preserving the natural integrity of the scenic area. Together, these elements depict a karst landscape characterized by diverse ecology, unique terrain, and a harmonious coexistence between humans and nature. (2) There is not a complete positive correlation between tourist sentiment bias and landscape diversity and vegetation coverage. Tourists pay more attention to the psychological perception brought by uniqueness, which is a bottom-up, surface to interior aesthetic perception path. The comprehensive emotional value of Huangguoshu is 14.35, and the emotional values are all positively distributed. The distribution density and extreme value of positive emotions are greater than those of negative emotions; (3) The emotional bias of tourists is directly related to visual sensitivity. The visual sensitivity of the Great Waterfall and Dishuitan areas is relatively high, mostly at I-II level sensitivity, and should be strictly protected to reduce development and avoid the destruction of their aesthetic value. However, the visual sensitivity of the Gaodang area is relatively low, and infrastructure should be optimized to scientifically improve aesthetic value. Finally, based on the current research achievements and shortcomings, this article proposes the trend and focus of future construction of aesthetic indicator systems and research on aesthetic value quantification methods, emphasizing the construction of a unified evaluation system. This requires not only the advancement of data collection, processing, and analysis technologies to accommodate the diversity and complexity of the data but also a deeper understanding and interpretation of the connotation of aesthetic value to ensure a comprehensive and dynamic evaluation. This is essential to provide scientific references for the conservation and development of natural beauty in WHKs.

## Supporting information

S1 File Model series code. (PDF)
