# Supplementary material for: Quantitative research on aesthetic value of the world heritage karst based on UGC data: A case study of Huangguoshu Scenic Area
Source: PLoS One. 2025 Feb 10;20(2):e0317304. doi: 10.1371/journal.pone.0317304 (PMC11809901; doi:10.1371/journal.pone.0317304)
Supplement: S1 File — (PDF) [file pone.0317304.s001.pdf]

```
import time
import cv2
import numpy as np
from PIL import Image
from segformer import SegFormer_Segmentation
if __name__ == "__main__":
    segformer = SegFormer_Segmentation()
    count           = True
    name_classes                                                                                                                    = ["_background_","waterfall","rivers","tree","house","forest","cave","farmland","shan","stone","food","people","cloth","car","ship","path","bridge","sky","light","rainbow","mask",]
    video_path      = 0
    video_save_path = ""
    video_fps       = 25.0
    test_interval = 100
    fps_image_path  = "img/street.jpg"
    dir_origin_path = "img/"
    dir_save_path   = "img_out/"
    simplify        = True
    onnx_save_path  = "model_data/models.onnx"
    if mode == "predict":
        '''
        seg_img = np.zeros((np.shape(pr)[0],np.shape(pr)[1],3))
        for c in range(self.num_classes):
            seg_img[:, :, 0] += ((pr == c)*( self.colors[c][0] )).astype('uint8')
            seg_img[:, :, 1] += ((pr == c)*( self.colors[c][1] )).astype('uint8')
            seg_img[:, :, 2] += ((pr == c)*( self.colors[c][2] )).astype('uint8')
        '''
        while True:
            img = input('Input image filename:')
            try:
                image = Image.open(img)
            except:
                print('Open Error! Try again!')
                continue
            else:
                r_image = segformer.detect_image(image, count=count, name_classes=name_classes)
                r_image.show()
    elif mode == "video":
        capture=cv2.VideoCapture(video_path)
        if video_save_path!="":
            fourcc = cv2.VideoWriter_fourcc(*'XVID')
            size                        =                        (int(capture.get(cv2.CAP_PROP_FRAME_WIDTH)), int(capture.get(cv2.CAP_PROP_FRAME_HEIGHT)))
            out = cv2.VideoWriter(video_save_path, fourcc, video_fps, size)
        ref, frame = capture.read()
        if not ref:
            raise ValueError("Failed to correctly read the camera (video), please pay attention to whether the camera is installed correctly (whether the video path is filled in correctly).")
        fps = 0.0
        while(True):
            t1 = time.time()
            ref, frame = capture.read()
            if not ref:
                break
            frame = cv2.cvtColor(frame,cv2.COLOR_BGR2RGB)
            frame = Image.fromarray(np.uint8(frame))
```

```
            frame = np.array(segformer.detect_image(frame))
            frame = cv2.cvtColor(frame,cv2.COLOR_RGB2BGR)
            fps  = ( fps + (1./(time.time()-t1)) ) / 2
            print("fps= %.2f"%(fps))
            frame = cv2.putText(frame, "fps= %.2f"%(fps), (0, 40),
cv2.FONT_HERSHEY_SIMPLEX, 1, (0, 255, 0), 2)
            cv2.imshow("video",frame)
            c= cv2.waitKey(1) & 0xff
            if video_save_path!="":
                out.write(frame)
            if c==27:
                capture.release()
                break
        print("Video Detection Done!")
        capture.release()
        if video_save_path!="":
            print("Save processed video to the path :" + video_save_path)
            out.release()
        cv2.destroyAllWindows()
    elif mode == "fps":
        img = Image.open(fps_image_path)
        tact_time = segformer.get_FPS(img, test_interval)
        print(str(tact_time) + ' seconds, ' + str(1/tact_time) + 'FPS, @batch_size 1')

    elif mode == "dir_predict":
        import os
        from tqdm import tqdm
        img_names = os.listdir(dir_origin_path)
        for img_name in tqdm(img_names):
            print(img_name)
            if img_name.lower().endswith(('.bmp', '.dib', '.png', '.jpg', '.jpeg', '.pbm', '.pgm', '.ppm',
'.tif', '.tiff')):
                image_path  = os.path.join(dir_origin_path, img_name)
                image       = Image.open(image_path)
                r_image     = segformer.detect_image(image,count=count,
name_classes=name_classes)
                if not os.path.exists(dir_save_path):
                    os.makedirs(dir_save_path)
                r_image.save(os.path.join(dir_save_path, img_name))

    elif mode == "export_onnx":
        segformer.convert_to_onnx(simplify, onnx_save_path)
    else:
        raise AssertionError("Please specify the correct mode: 'predict', 'video', 'fps' or
'dir_predict'.")
```
